# Supplementary material for: Influence of malaria, soil-transmitted helminths and malnutrition on haemoglobin level among school-aged children in Muyuka, Southwest Cameroon: A cross-sectional study on outcomes
Source: PLoS One. 2020 Mar 30;15(3):e0230882. doi: 10.1371/journal.pone.0230882 (PMC7105131; doi:10.1371/journal.pone.0230882)
Supplement: S1 File — (PDF) [file pone.0230882.s001.pdf]

QUESTIONNAIRE

A. PUPIL'S IDENTITY

1 Name of school: ____

2. Sex of pupil: male: ______ Female: _______ 3. Age:_________ 4. Class:_______

5. Place of residence: _________________

B. DEMOGRAPPHIC INFORMATION

1. Level of education of parent a. Primary b. Secondary c. High school d. University e. Others

2. Parent/guardian occupation. Farmer_____ Business_____ Others____

C. MALARIA CONTROL

3. What type of house? a. Block b. Brick c. Plank d. Others

4. Do you have fever now? Yes__ No___

5. When did you have fever? Yesterday ___ Last week ___Last month___

6. Have you taken malaria medicine within the last two weeks? Yes____ No____

7. Do you have a. Headache b. Joint pains?

8. When you are sick, do you take medicine? Yes ____ No____

9. Do you sleep under the mosquito net? Yes ____ or No___

10. How often do you sleep under the mosquito net? Every day ___Sometimes ___Never____

11. Do you have bushes around your house? Yes __ No___

D. HELMINTH CONTROL AND PREVENTION MEASURES

12. Have you taken any worm medicine in the last two months? Yes___ No____

13. Do you take any herbs for treatment of worms? Yes____ or No_____

14. If yes, what type of herbs? _______________

15. What type of floor do you have in your house? Earthen floor_____ Cemented floor______

16. Do you have a toilet? Yes______ No_____

17. Which type of toilet? Internal______ or External______

18. How often do you clean your toilet? a. Always b. Sometimes c. Never

19. Do you defecate in the toilet? Yes___ No____

20. How often do you wash your hands after defecating? Always______ Sometimes_____ Never___

21. Does your school have a toilet? Yes_____ No______

22. Where do you carry water for drinking? Tap____ Stream_____ Well_____ Other____

23. Do you always wear shoes? Yes ____ No____

24. How often do you walk barefooted? Always______ Sometimes_____ Never______

25. How often do you go to the farm? Everyday_____ Every week ______

26. Do you experience stomach ache? Yes______ No______

27. Do you experience a. Diarrhoea b. Vomiting c. Others (specify) _________?
