# Supplementary material for: Influence of malaria, soil-transmitted helminths and malnutrition on haemoglobin level among school-aged children in Muyuka, Southwest Cameroon: A cross-sectional study on outcomes
Source: PLoS One. 2020 Mar 30;15(3):e0230882. doi: 10.1371/journal.pone.0230882 (PMC7105131; doi:10.1371/journal.pone.0230882)
Supplement: Attachment — Submitted filename: Response to Reviewers 2.docx [file pone.0230882.s004.docx]

Dear Editor

Many thanks for your email of 29 January, 2020 providing comments on our manuscript and inviting us to submit a second revision. We have now prepared a revised manuscript reflecting the suggestions made by the Editor for consideration. Below we have provided point-by-point response to the concerns and comments detailing the changes we have made in response to each point raised. The changes made to the manuscript based on the comments and concerns of the Editor and reviewers have been tracked in the revised manuscript for appraisal. We would like to thank the Editor and reviewers for taking the time to read our manuscript again and to provide thoughtful and helpful comments for revision. We believe the revised manuscript represents a significant improvement on the original.

We look forward to hearing from you in due course

Irene Sumbele (on behalf of all authors)

See Authors’ responses to Editor and Reviewer’s comments in the attached document.

**Responses to Editor and Reviewers’ Comments and Concerns**

**Title: Haemoglobin level as an indicator of health status of school-aged children in Muyuka, Southwest Cameroon: Influence of malaria parasites, soil-transmitted helminths and malnutrition**

**Authors**

Irene Ule Ngole Sumbele^1, 3*^, Ayeah Joy Nkain^1#a^, Teh Rene Ning**^1^**, Judith Kuoh Anchang-Kimbi^1^, Helen Kuokuo Kimbi^1,2^

**Version: 3**

Date: 12 February, 2020.

Authors’ responses to Editor and reviewers’ comment over leaf.

**Response to Comments and Concerns of the Editor**

**Academic Editor’s Comments:**

**Dear authors,**

**Thank you for submitting your revised manuscript to PLoS One. This version has addressed the reviewers’ comments; however, corrections remain and additional comments should be addressed before this manuscript can be accepted. Please consider the following:**

**1- In laboratory procedures section, it is mentioned that malaria parasite density per μL of blood was determined; however, information on this variable was not provided in the manuscript. Using the term “malaria parasitaemia” in the current manuscript without providing these results is unclear and can be misleading. It can be replaced with malaria infection. Moreover, it is interesting to examine the association between the level of parasitaemia (parasite density) and Hb level and anaemia prevalence as well, and this should be incorporated into the results & discussion of this manuscript.**

The authors apologize for not detailing the report of the malaria parasite (MP) density in the manuscript. The relationship between MP density and anaemia was reported in Table 4 earlier but the categories were not clearly defined as shown in the highlight. We have included the definition of low, moderate and high parasite densities in the method and as a foot note in the Table for clarity. As concerns malaria parasitaemia, the mean MP density and range has been included in Table 2 and the relationship between MP and Hb level reported in Fig 2. No statistically significant association was observed between MP density and anemia likewise between Hb level and MP even though a seemingly decreasing trend was revealed in Figure 2. This was taken into consideration during the discussion earlier therefore, the discussion was based on the prevalence of MP and AR in association with the other conditions rather than density alone. Hence, the authors don’t see the need for more discussion on malaria parasite density and Hb level in this instance. Furthermore, the relationship between Hb level and malaria parasitaemia was discussed further in Lines 508-511.

2- **The attributable risk (AR%) was mentioned in statistical analysis section (please remove details on formula used) and in discussion and conclusion; however, results were not provided in results section.!**

The authors wish to disagree on this point. The results on attributable risk were presented in lines 349 to 350 in the results as indicated in the highlight that is why they were discussed. In the context of the study, this calculation is necessary to show the relative contribution of each factor to the occurrence of anaemia.

3- **I suggest change the title. Presentation of your results and your conclusion are not compatible with the title “Haemoglobin level as an indicator of health status of ….”. Basically, the title can be “Influence of malaria, soil-transmitted helminth infections and malnutrition on haemoglobin level and anaemia prevalence among school-aged children in Muyuka, Southwest Cameroon”. You may think about alternative titles.**

The title has been revised to “Influence of malaria, soil-transmitted helminth infections and malnutrition on haemoglobin level among school-aged children in Muyuka, Southwest Cameroon: a cross sectional study on outcomes. We the authors believe this is more embracing. Placing haemoglobin level and anemia prevalence on the title is kind of odd. Anaemia is an outcome of these infections. It as well embraces the health status.

**4- Tables format should be improved and edited for consistency and English. For instance:**

The tables have been edited and formatted as shown in the tracked changes.

**4.1. Table 5: change columns of total to “overall mean (SD) Hb level” and this column can be placed after prevalence o anaemia column. P-value for sex can be placed after the columns of mean Hb for sex and p-value for age groups will be accordingly after the columns of age. Indicate the use of t-test and ANOVA in the footnotes.**

The corrections have been effected as requested. See tract changes for the corrections.

**4.2. Table 3: the data are % (n) but it is indicated as (n) only.**

Since the table is reporting prevalence, we the authors thought it wasn’t necessary to indicate percentage again as the unit of measurement. However, we have included it as requested. See tracked changes.

**4.3. Table 6: please report important necessary results only for the output of MLR analysis; collinearity statistics and correlation coefficient columns can be removed. Please read about reporting such results in scientific papers or refer to previous literature. For instance, provide B value, standard error, 95% CI and p value for the included variables.**

The Table was a composite table of two different out puts. However, the table has been revised as requested to include the B value, standard error and 95% CI for variables calculated.

**5- Figure 2 can be removed. Renumber the figures.**

Figure 2 has been deleted and the results written out for clarity. A new Fig 2. that shows the Hb level and malaria parasite counts in the population has been produced in response to editors request of evidence of parasitaemia in the manuscript.

**6- What is URIT-12 haemoglobin meter? Company, City, Country, and cite reference for its validity.**

The specification has been included “URIT Medical Electronic co., Ltd, London, United Kingdom)”.

**7- Follow first appearance rule; for instance, spell out the MUAC in abstract and then add the abbreviation between brackets. Follow this in the entire text and tables. In tables, define the abbreviations in the footnotes.**

The corrections have been effected as indicated in the tracked changes.

**8- Moreover, the manuscript needs extensive editing by a native English ‎speaker. Please ‎note that poor English may ‎ultimately be a reason to reject the ‎manuscript.**

The manuscript has been extensively edited by a native English speaker as requested. See the tracked changes for the copy editing.‎ ‎

**[Note: HTML markup is below. Please do not edit.]**

**Reviewers' comments**

The comments in the file attached by reviewer 2 had been adequately addressed in the first round of review process as indicated by the reviewer.
